# Alternative Exercise Technologies to Fight against Sarcopenia at Old Age: A Series of Studies and Review

**DOI:** 10.1155/2012/109013

**Published:** 2012-02-20

**Authors:** Wolfgang Kemmler, Simon von Stengel

**Affiliations:** Institute of Medical Physics, Friedrich-Alexander University, Erlangen-Nürnberg, Henkestraße 91, 91052 Erlangen, Germany

## Abstract

The most effective physiologic mean to prevent sarcopenia and related muscle malfunction is a physically active lifestyle, or even better, physical exercise. However, due to time constraints, lack of motivation, or physical limitations, a large number of elderly subjects are either unwilling or unable to perform conventional workouts. In this context, two new exercise technologies, whole-body vibration (WBV) and whole-body electromyostimulation (WB-EMS), may exhibit a save, autonomous, and efficient alternative to increase or maintain muscle mass and function. Regarding WB-EMS, the few recent studies indeed demonstrated highly relevant effects of this technology on muscle mass, strength, and power parameters at least in the elderly, with equal or even higher effects compared with conventional resistance exercise. On the contrary, although the majority of studies with elderly subjects confirmed the positive effect of WBV on strength and power parameters, a corresponding relevant effect on muscle mass was not reported. However, well-designed studies with adequate statistical power should focus more intensely on this issue.

## 1. Introduction

The negative change of muscle mass from maturity to senescence and the corresponding loss of functional capacity are of high clinical significance [1, 2]. The most physiologic means to fight this decline of muscle mass and function is a physically active lifestyle or even better, physical exercise [3]. Indeed, a plethora of exercise studies (review in [4–7]) proved favorable changes of muscle mass, power, and strength parameters. However, to realize relevant changes of muscle mass, strength, and power, exercise has to be performed regularly with moderate exercise frequency (≥2 sessions/week) and moderate to high levels of intensity [6, 8]. Due to physical limitations or to lack of motivation, a large number of elderly subjects obviously seem to be either unable or unwilling to perform (intense) corresponding resistance exercise programs.

In this context, exercise technologies that increase the impact of low-level exercise on the musculoskeletal system are of high relevance. Recently, two promising new technologies that primarily focus on the aim to increase endogenous loading by external strain were presented. One of both technologies focuses on the increased response of muscular activity when exposed to vibration stimuli (whole body vibration (WBV) training); the other focuses on the stimulation of large muscle groups by electric stimuli (whole-body electromyostimulation (WB-EMS) training).

The purpose of this paper is to summarize our corresponding results and experiences in this promising area and to generally review the effect of both technologies on muscle mass and function in older adults.

## 2. Whole-Body Electromyostimulation

Electromyostimulation is known as an established technology primarily practiced as a local, passive, either more therapeutically, or more athletic application [9]. Briefly, during EMS, impulses are transmitted through electrodes on the skin close to the muscles in order to stimulate. These impulses cause involuntary contractions of the muscles and thereby preferentially recruit fast-twitch fibers that are predominantly affected by age-induced muscle atrophy [10].

The recently presented whole-body electromyostimulation technique focuses on the stimulation of large segments (all main muscle groups; 2.7 m^2^ area) during slight endogenous movements or exercises [11]. Beside this simultaneously, activation of large, areas another strong point of WB-EMS is the possibility to stimulate each (main) muscle group separately and differentially. Along with the maintenance or increase of muscle mass and corresponding resting metabolic rate (RMR) [12], this acute overall activation of fibers with high-energetic demands leads to a significant increase of energy expenditure [11] with impact on energy balance and overweight/adiposity [13]. Finally, although corresponding dose-response studies are not available, the recommended exercise frequency of WB-EMS is rather low, which was in line with the unwillingness of most (elderly) subjects to spend a lot of time for exercising [14]. Taken together these pros, WB-EMS was promising to address the frail elderly subjects that are unable (or unwilling) to participate in conventional resistance exercise programs with an exercise frequency and intensity being adequate to promote relevant positive effects on muscle mass.

However, although the majority of classical EMS studies using locally and passively applied electromyostimulation improved (neuro-)muscular capacity [15–18], only one study observed significant positive changes (however, no significant “effect” (as defined as differences between WB-EMS and control concerning intragroup change from baseline to follow-up test) was observed) of thigh muscle mass in frail elderly with COPD [19]. Beside the vague evidence concerning EMS-induced changes of muscular parameters, data about the acceptance and feasibility of this technology in this cohort are also scarce.

Thus, the purpose of the two studies presented here was to assess the effect of WB-EMS on strength parameters and body composition/muscle mass in elderly subjects. Both studies are briefly described and discussed; for a more detailed review, the reader is kindly referred to the original publication.

## 3. Methods

Both studies were approved by the Ethics Committee of the University of Erlangen (Ethik-Antrag 3777 and 3876). All study participants were informed of the experimental risk and gave written informed consent. Exclusion criteria for both studies were epilepsy, cardiac pacemaker, grave circulatory disorders, abdomen/groin hernia, tuberculosis, cancer, grave neurologic disturbances, inflammatory diseases, bleeding tendencies, medication, or diseases affecting muscle metabolism.

**Figure 1:**
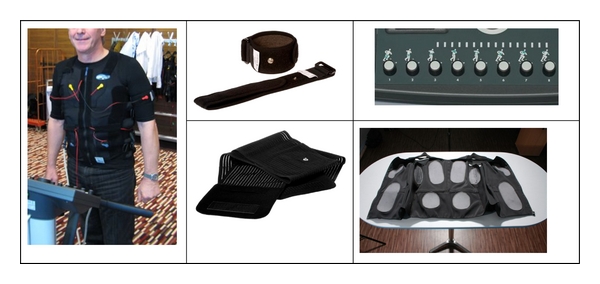
WB-EMS equipment (miha bodytec, Augsburg, Germany).

EMS exercises were performed with WB devices of miha bodytec (Augsburg, Deutschland). The WB-EMS equipment (Figure 1) enables the simultaneous activation of 16 regions (e.g., upper legs, upper arms, backside, abdomen, chest, lower back, upper back, and shoulder; total size of electrodes: *≈*2650 cm^2^) with dedicated intensity.

### 3.1. Training and Electromyostimulation (TEST-I) Study: Effect of WB-EMS in Well-Trained Postmenopausal Females with Adjuvant Exercise Training

The purpose of the TEST-I study [12] was to determine the adjuvant effect of WB-EMS on body composition and strength/power parameters in a cohort of exercising elderly females.

Thirty postmenopausal females (65 ± 5 yrs, BMI: 24.7 ± 4.1 kg/m^2^) were randomly assigned either to a control group (CG, *n* = 15) that maintained their general exercise program of twice 60 mins/week or to a WB-EMS group (*n* = 15) that additionally performed a 20-minute WB-EMS exercise program every five days for 14 weeks. WB-EMS group-sessions (three participants at three EMS devices supervised by one research assistant) consisted of 15 dynamic exercises for all main muscle groups using a small range of movement (ROM) to prevent adaptations by the physical exercise per se. Two different bipolar protocols, one with 85 Hz of intermitted stimulation (4 s exercise–4 s rest) and one with continuously implicated stimulation (7 Hz), each of 10 min long, were carried out. Participants were carefully instructed by research assistances how to perform the exercises and how to adjust the intensity of the current. Subjects were asked to exercise at a moderate to high rate of perceived exertion.

Muscle mass was indirectly assessed by resting metabolic rate (RMR) as determined by indirect calorimetry [20] (Oxycon mobile, Conshohocken, USA). Body fat was determined by skinfold measurement (Lange, Cambridge, USA) at eleven anatomical sites and by Bio-Impedance Analysis (Biospace, Seoul, Korea). Further, waist circumference was determined.

Maximum strength of the trunk extensors and flexors was assessed with a Schnell M3 (Peutenhausen, Germany) isometric tester. Leg extensors strength (leg press) and leg power as determined by a countermovement jump were assessed with a force-measuring plate (MTD-System, Neuburg v. Wald, Germany).

Compliance with the WB-EMS regime was determined by utilizing the attendance recorded by research assistants and using questionnaires.

After 14 weeks of exercise, significant positive effects (i.e., changes within WB-EMS versus control) were observed for body fat (sums of skinfold: WB-EMS: −8.7 ± 7.1 (*P* = .001) versus CG: +1.2 ± 5.9% (n.s.); intergroup difference: *P* = .001), waist circumference (−2.3 ± 1.8 (*P* = .001) versus +1.0 ± 1.7% (n.s.); *P* = .001), strength (+6.6–9.9% (*P* < .001) versus −4.5–6.3% (n.s.); *P* < .006), and power (+8.7 ± 5.5 (*P* = .001) versus −1.2 ± 6.8% (n.s.); *P* = .001) parameters, while the effect on RMR did not reach a statistically significant level (−0.2 ± 8.1 (n.s.) versus −5.3 ± 8.7% (*P* = .04); *P* = .09).

Concerning the attractiveness of the WB-EMS intervention, no subject dropped out during the EMS period, and attendance rate of the EMS intervention was 98% compared with a corresponding rate of 80% (no differences between WB-EMS and CG) within the joint general exercise training. All but one participant stated they would like to continue WB-EMS exercise training.

Taken together, in this cohort of elderly females with long year participation in high-intensity resistance exercise protocols [21], WB-EMS exercise generated impressive changes of strength, power, and, although nonsignificant, parameters related to free-fat mass (RMR; [20]) compared with a rather active control group.

However, there are some limitations that complicated the interpretation of the findings. (1) Although RMR was closely correlated to fat free mass, more sophisticated technologies to assess body composition (MRT, DXA) may be more accurate to determine changes in body composition. (2) The choice to recruit a cohort of trained females that were able and willing to strictly adhere our EMS protocol was important to ensure the realization of the prescribed exercise intensity and volume; however, it can be presumed that the corresponding effect on muscle mass and strength might be more pronounced in sedentary untrained females. (3) Further, the effect (i.e., difference between within-group changes in WB-EMS versus control) might be more distinct if the control group were inactive.

In summary, beside relevant effects on fitness and fatness parameters, a high acceptance and feasibility of whole body EMS training exercise was verified in this group of trained elderly females.

### 3.2. Training and Electromyostimulation (TEST-II) Study: Effect of WB-EMS in Untrained Elderly Males with the Metabolic Syndrome

The purpose of the TEST II study [13] was to validate the effect of WB-EMS on body composition, metabolic syndrome, and physical capacity parameters in untrained males of 65–75 years old. To account for the weak points of TEST-I, more sophisticated techniques of body composition measurement were used. Further, we compared our untrained verum group with a control group that was additionally blinded by performing very easy exercises on a vibration plate.

28 sedentary males (69 ± 3 y, BMI: 27.8 ± 4.5 kg/m^2^) with the metabolic syndrome according to the International Diabetes Federation [22] were randomly assigned either to a WB-EMS group that performed a mixed endurance/resistance exercise protocol or to a control group that performed slight movements on vibration platforms (Fibrafit, Solms, Germany), both over a period of 14 weeks. The WB-EMS group exercised 30 min every 5 days. The session was structured in an endurance sequence (15 min at 70–85% Hfmax on a cross-trainer) with continuously applied stimulation (bipolar, 85 Hz) and 15 min of “resistance” exercises for all main muscle groups with slight movements and a small range of movement (see test I; intermitted 4 s exercise—4 s rest, bipolar, 85 Hz). Research assistants supervised each session and instructed participants how to perform the exercises and encouraged subjects to exercise at a moderate to high rate of perceived exertion. Comparably to TEST I, the intensity of the current as the central parameter was regularly adjusted within the session to ensure a continuous moderate to high exposure.

To blind participants of the control group a slight “exercise program” on vibration plates (18 min, Frequency: 30 Hz; amplitude: 1.7 mm) was carried out. Using video animation preferentially stretching exercises, standing with one leg beside the plate, whereas the stretched leg was positioned on the plate, were performed. Additionally, some both-legged slight movements should be performed on the plates with a perceived exertion of low to moderate. After an initial introduction of two to three sessions, vibration training was not completely supervised but regularly controlled by research assistants.

Body composition was assessed by dual energy X-ray absorptiometry (Hologic QDR 4500 Discovery upgrade, Bedford, MI, USA), using a whole-body scan and a regional analysis of the abdominal “region of interest.” Further waist circumference and blood parameters were assessed to determine changes of the metabolic syndrome.

Strength and power were determined comparably to the TEST-I study (see above). Additionally, we evaluated changes in aerobic capacity. A stepwise test on a cross-trainer was performed to a voluntary maximum exertion. Every 3 min, resistance was increased by 20 Watt at a constant speed of 120–130 rpm. VO_2_ max was measured breath by breath using an Oxycon mobile (Viasys, Conshohocken, PA, USA) open spirometric system.

After 14 weeks of exercise, intention to treat analysis determined significant effects (intergroup differences) for total muscle mass (WB-EMS: +298 ± 993 g (n.s.) versus CG: −600 ± 838 g (*P* = .04), intergroup difference: *P* = .020), appendicular skeletal muscle mass (ASMM: +249 ± 444 g (n.s.) versus −268 ± 664 g (n.s.), *P* = .028), total fat mass (−1.35 ± 0.88 kg (*P* = .001) versus −0.42 ± 0.85 kg (n.s.), *P* = .012), abdominal fat mass (−252 ± 196 g (*P* = .001) versus −52 ± 128 g (n.s.); *P* = .006), and waist circumference (−5.7 ± 1.8 (*P* = .001) versus −3.0 ± 2.0 cm (*P* = .006), *P* = .001), while no statistical significant difference (*P* = .918) was observed for body mass.

Isometric strength of the leg extensors (leg press) increased by +15 ± 11% (*P* = .001) in the WB-EMS group and was maintained (+3 ± 4%, n.s.) in the CG. In parallel, leg power increased by 10 ± 7% (*P* = .001) in the WB-EMS and nonsignificantly decreased by −0.5 ± 6% in the CG. Corresponding changes were determined for relative (mL/min/kg) VO_2_ max (WB-EMS: +15 ± 9%, *P* = .001 versus CG: +3 ± 3%, n.s.). All these effects were highly significant (*P* = .001). However, with the exception of waist circumference, no further significant effects on metabolic syndrome parameters (blood lipids, glucose, and blood pressure) were verified.

In TEST II, we clearly demonstrated the effectiveness of WB-EMS to increase muscle mass and reduce fat mass, both on a relevant and statistically significant level. This factor is of importance because “sarcopenic obesity” [23, 24], that is, the parallel development of excessive overweight and the reduction of muscle mass and function, may potentiate their deleterious effects on physical disability, morbidity and mortality. However, only a minority of interventions were able to positively impact both factors. While energy restriction leads to significant reductions of fat free mass [25, 26], there is some evidence that physical exercise positively impacts both systems [26], although the favourable effect on fat reduction was less pronounced than under energy restriction [25]. However, to our best knowledge, we are not aware of conventional exercise studies that reported comparably favourable changes of body fat and free fat mass. The results are remarkably especially considering the short duration, the rather unspectacular obesity status (2 subjects in the WB-EMS group and 3 subjects in the CG group with BMI >30 kg/m^2^) of the participants, and the “nonsedentary” vibration control group with their favourable effects on body fat parameters.

### 3.3. Summary of Whole-Body Electromyostimulation (WB-EMS)

Whole-body Electromyostimulation is a new promising exercise technology that may be able to motivate subjects unwilling or unable to perform conventional exercise programs with impact on body composition and strength/power development.

Beside its clinical effectiveness, the strong points of this alternative exercise technology are (1) the possibility, in contrast to a passive/static application, to perform dynamic exercises, while electromyostimulating ensures a harmonically increase of strength and power throughout the selected range of motion and thus has also an implication on functional capacity [27], (2) the low frequency and duration of the EMS application allow to reach subjects with time constraints. Even more relevant, (3) the mobility of the system that enables an ambulatory implementation in nursing homes, residential care, or as an application operated by personal trainers or nurses at home, and (4) due to the low duration of a session (20 min), ambulatory EMS-programs were able to exercise a reasonable amount of participants per time unit.

However, some limitations may actually prevent a broad application of this technique to exercise elderly subjects prone to frailty. (1) Basically, WB-EMS is an expensive exercise technology (cost for one WB-EMS device including equipment *≈*10.000 €). Further, if applied on a daily base current costs due to abrasion of the equipment may average 1000 € per year per device. Additionally, labor costs due to the actual need of a supervisor (see below) relevantly increase current costs. However, based on our experience of 4-year WB-EMS application with three EMS devices for three contemporaneously exercising subjects, one supervisor, 3-4 hours of application per day, and no additional room rental and incidental expenses, an overall 25 min noncommercial EMS session should be calculated with 5–7 €/participant. However, taking into account the lower frequency of EMS-application compared with conventional exercise programs (1-2 versus 2-3 sessions/week [28]), costs are well within the range of other effective interventions.

(2) Although video-animated guidance and feedback systems may ensure an independent, efficient and safe EMS application, elderly subjects may shy at starting an unaffiliated intervention with this technology. As mentioned, actually the WB-EMS procedure requires the supervision and continuous presence of a trainer; thus, for a broad and economic implementation of this technology, it would be preferable to simplify the WB-EMS procedure.

(3) Although we are not aware of relevant side effects, WB-EMS may not be acceptable for all elderly subjects. Beside the contraindications given also for conventional EMS devices (epilepsy, cardiac pacemaker, grave circulatory disorders, abdomen/groin hernia, tuberculosis, cancer, grave neurologic disturbances, inflammatory diseases, bleeding tendencies, skin irritations, wounds, and burns), the high energetic demands during EMS which are characterized by a significant increase of acute energy expenditure may be problematic for subjects with severe metabolic disturbances.

### 3.4. Whole-Body Vibration

In the past few years, WBV has been proposed as a mild approach to counteract sarcopenia in the elderly. Standing on an oscillating platform induces a reflectory enhanced response of the leg and postural muscles via the so-called “tonic vibration reflex” [29]. This reflectory response might be the key to long-term functional and structural neuromuscular adaptations which were observed in several studies. However, the potential of WBV to induce muscular strength is still unclear [30]. Study results suggest that preferentially untrained or older individuals with low fitness levels benefit from WBV. According to that, contrarily to studies with young individuals, the majority of studies with elderly report positive results with respect to parameters related to sarcopenia [31]. Gains in maximum leg strength were reported in postmenopausal women [32–36], elderly men [37], or mixed geriatric cohorts [38, 39] after long-term WBV training. Correspondingly, increases in jumping height [33, 37, 40] and muscle power [38, 41, 42] were observed in older individuals. In studies that implemented an “active” control group that performed resistance [33–35] or “fitness” exercise training [37], WBV and conventional exercise programs resulted in comparable gains in maximum leg strength and power.

No increase of lean body mass, as assessed by DXA, was induced by WBV exercise [35, 43, 44] or by adding WBV to resistance training [45, 46] indicating that predominantly neuronal mechanisms contribute to the observed strength and power gains. However, local muscle mass changes (thigh muscle cross-sectional area) which were determined using CT [32, 37] suggest rather site-specific morphological effects.

The neuromuscular effects and the underlying mechanisms of WBV exercise are still largely unclear. The purpose of the ELVIS I and II trial (Erlangen longitudinal vibration study I) was to assess the effect of WBV on bone mineral density, body composition/muscle mass, and neuromuscular performance in elderly women. Both the studies, however, had different aims: in the ELVIS I study, we examined wether the effect of multipurpose exercise can be enhanced by whole-body vibration (WBV). In the ELVIS II trial, we determined the effect of an isolated WBV training on different WBV devices (vertical versus rotational). In this contribution, the effects on parameters related to sarcopenia will be focused. For more detailed information, the reader is kindly requested to read the original publications [36, 43, 46, 47].

**Methods**

Both studies were approved by the Ethics Committee of the University of Erlangen (Ethik Antrag 3354 and 3693). All study participants gave written informed consent before study start. Exclusion criteria for both studies were (1) diseases or medication affecting bone metabolism, (2) diseases or medication affecting neuromuscular performance, (3) implants of the lower extremity or spine, (4) eye diseases affecting retina, and (5) low physical capacity (<50 W).

### 3.5. Erlangen Longitudinal Vibration Study (ELVIS I)

In the ELVIS I trial, a randomized controlled 18-month interventional study, we examined if whole-body vibration (WBV) could enhance the effect of a conventional multifunctional exercise training program consisting of aerobic dance, resistance, flexibility, and coordination training with respect to neuromuscular performance and body composition. 151 postmenopausal women (68.5 ± 3.1 y) were randomly assigned to a (1) training group (TG), a (2) training group including vibration (VTG), or a (3) wellness-control group (CG). TG and VTG performed the same training program twice weekly (60 min) consisting of aerobic and strength exercises with the only difference that leg strength exercises sequence (15 min) was performed with (VTG) or without (TG) vibration. The training sessions consisted of (1) 20 min of aerobic dancing, (2) 5 min of general coordination and balance training, (3) 20 min functional gymnastics, dynamic (using elastic belts (Thera-Band^,^ Hadamar, Germany)) and isometric strength exercises, and stretching and (4) 15 min of dynamic leg strengthening exercises performed on vibration platforms. In VTG, the plates (Vibrafit, Solms, Germany) vibrated at a frequency of 25 Hz and an amplitude of 1.7 mm, whereas the plates were switched off in TG. Three exercises (heel rises, one-legged deep squat, and leg abduction) were performed in a circuit mode twice. One minute of leg-strength exercise (*≈*12 reps at 60–70% 1RM) was intermitted by one minute of stretching exercise. In course of the study, vibration frequency (30, 35 Hz) and exercise intensity were increased (by modifying exercises). To ensure compliance and in order to blind participants, CG performed a low-intensity “wellness” program with different aims, which should be below the threshold for structural adaptations.

At baseline and after eighteen months, body composition was determined using dual X-ray absorptiometry (DXA) (QDR 4500A, discovery upgrade, Hologic, Bedford, MA). Maximum isometric leg strength was determined on a static leg press using a force-measuring plate. Leg power was measured by countermovement jumps (CMJ) via force measuring plate. Maximum strength of the trunk flexors and extensors was assessed in an isometric mode using a Schnell M3 device (Peutenhausen, Deutschland).

In both the training groups, lean body mass (LBM) was positively affected (VTG: 0.4 ± 1.2 kg, n.s. versus TG: 0.6 ± 1.5 kg, *P* = .01), whereas only the change in the TG was significant (*P* = .03) compared to the CG (−0.2 ± 1.8 kg).

Maximum leg strength significantly (*P* < .001) increased in both the exercise training groups (VTG: +16 ± 27% versus TG: +12 ± 20%), but only the difference between VTG and CG (+5 ± 21%, n.s.) was significant (*P* = .  02). Although both exercise training groups showed positive results for trunk flexion strength (VTG: +16 ± 28%, *P* = .001 versus TG 8 ± 27%, n.s.), a significant between-group difference (*P* = .03) compared to the CG (+2 ± 29%, n.s.) was observed for the VTG only. Also, both the training groups significantly (*P* < .01) gained trunk extension strength (VTG: +6 ± 17%; TG: +7 ± 18). Further, compared to the CG (−4 ± 13%, n.s.), both changes were statistically significant (*P* = .001).

Leg power significantly increased in the VTG only (+8 ± 13% versus TG: +4 ± 18% versus CG: +2 ± 11%), however, intergroup differences were not significant (*P* > .25). Taken together, the effects of the multipurpose exercise program were not enhanced by WBV. For neuromuscular performance, there was just a tendency in favour of the exercise training protocol using vibration.

### 3.6. Erlangen Longitudinal Vibration Study (ELVIS II)

Isolated WBV might be a potent strategy to counteract sarcopenia especially for people who are not willing or able to perform a time-consuming and strenuous strength training program. Thus, the philosophy of the ELVIS II trial was to exclusively perform WBV exercise to determine the potential of isolated WBV as an alternative to conventional exercise training programs. However, the most effective vibration type along with the most favourable vibration parameters (i.e., frequency, amplitude, etc.) and the longitudinal training strategy to maximize neuromuscular effects is still unidentified. With respect to the construction of vibration plates, there are two principally different systems: (1) plates which rotate around a fulcrum, producing alternating forces to the left and right foot; (2) devices in which the whole plate vibrates vertically producing forces on both feet simultaneously.

In the ELVIS II trial, a randomized controlled 12-month interventional study, we determined the effect of WBV training using two different WBV devices (rotational: Qionic, Burtenbach, Germany versus vertical: Vibrafit, Solms, Germany). 108 postmenopausal women (65.8 ± 3.5 y) were randomly allocated to (1) vibration training on rotational plates (RVT; 12.5 Hz, 12 mm) or (2) on vertically vibrating plates (VVT; 35 Hz, 1.7 mm). Both groups performed a WBV training consisting of 3 sessions/week, lasting 15 min each. In the 15 min training program, seven one- or two-legged dynamic leg strengthening exercises were performed on the plates in standing position, each lasting 90 seconds: two-legged squat, two-legged dynamic squats including heel rises, leg abduction, and one-legged squats, one-legged squat including hip flexion of the contralateral side, followed by exercise one and two again. Strengthening exercises were intermitted by stretching exercises of 40 s. Both vibration protocols resulted in a similar acceleration (about eight times earth gravitational force (8 g)).

In the first three sessions, women were introduced to the vibration devices and the exercises. In the following, the women performed the WBV training accompanied by video-animation on their own, however, proper completion of the exercises were regularly checked by an instructor.

Comparably to the ELVIS I, body composition was determined using DXA, and maximum isometric strength and power were determined for lower extremity, maximum strength for trunk flexion and extension (see above).

After 12 months of WBV, the feasibility of the video-instructed training was good (dropout: RVT < 20%; VVT < 10%; attendance rate 70% in both groups). Both VT groups significantly gained maximum leg strength (RVT: +27 ± 22%; VVT: +24 ± 34%) compared to CG (+6 ± 20%) (*P* < .001), whereas the moderate gains in power in both training groups were not significant compared to CG. There was a significant gain in maximum trunk flexion (VVT: 12 ± 29%; RVT: 12 ± 21%; KG: −6 ± 22%) (*P* < 0.01), but not in extension strength; However, no significant changes were observed for LBM.

In summary, the ELVIS I and II trial demonstrated a positive effect of WBV exercise with or without additional conventional exercise on maximum strength, however the effect on lean body mass as determined by DXA was negligible.

### 3.7. Summary of Whole-Body Vibration

Taken together, the majority of WBV studies with elderly demonstrated positive changes of muscle strength or power. However, the reported gains in maximum strength (about 15% on average with the exception of Machado et al. [32]: 38% and von Stengel et al. [46]: 25%) were rather small compared to the results of studies which performed a specific resistance training regimen [48]. Contrarily to specific resistance training protocols [49, 50], WBV did not show relevant effects on LBM, indicating neuronal mechanisms as main mechanism for increasing strength or power. Studies that determined regional muscle mass via CT further suggest rather site-specific structural effects of WBV.

Due to orthopaedic and cardiac limitations or simple aversion, many people are either unwilling or unable to perform vigorous resistance training programs. For those frail people, WBV may be an alternative to counteract sarcopenia. Given the feasibility, low demands on manpower and high time flexibility, a video-based WBV training, like we used in the ELVIS II study, has the potential for large-scale implementation in different institutions.

Though no WBV vibration-related adverse effects were determined in current studies, there is still no knowledge about a potential threshold beyond which WBV may overload bone or other tissues causing adverse effects, especially in elderly people with reduced adaptability of the musculoskeletal system. The knowledge about the optimum intensity (dependent on vibration type, amplitude, acceleration, duration, and standing position (i.e., knee angle, heels versus forefoot)) which effectively triggers adaptations but prevents overload is also still scarce.

## 4. Conclusion

There is a good body of evidence that WB-EMS and somewhat restricted WB Vibration are effective to increase muscle mass and functional capacity in the elderly subject.

Beside their clinical effectiveness, the main advantages of both technologies compared with conventional exercise are in summary as follows: (1) the mode of operation of the devices that increases subjects' voluntary loading to a higher and hence more effective level. Thus, subjects unwilling or unable to exercise with adequate (high) exercise intensity may benefit from the adjuvant application of WB-EMS or WBV although exercising at an otherwise below threshold level, (2) the time-saving effect of both technologies, WBV and particularly WB-EMS, compared with conventional resistance exercise programs with multiple sets and repetitions for each muscle group, (3) the option to exercise flexible on one's own authority, and (4) the general exclusiveness and attractiveness of new exercise technologies compared with apparently “old-fashioned weight lifting” programs.

Thus, both technologies may be attractive especially for subjects otherwise unable or unwilling to exercise conventionally and will be therefore a promising option to increase subjects' physical activity up to a level that fights sarcopenia.
